# The TREC 2004 genomics track categorization task: classifying full text biomedical documents

**DOI:** 10.1186/1747-5333-1-4

**Published:** 2006-03-14

**Authors:** Aaron M Cohen, William R Hersh

**Affiliations:** 1 Department of Medical Informatics and Clinical Epidemiology, School of Medicine, Oregon Health & Science University, 3181 S.W. Sam Jackson Park Road, Mail Code: BICC, Portland, Oregon, 97239-3098, USA

## Abstract

**Background:**

The TREC 2004 Genomics Track focused on applying information retrieval and text mining techniques to improve the use of genomic information in biomedicine. The Genomics Track consisted of two main tasks, ad hoc retrieval and document categorization. In this paper, we describe the categorization task, which focused on the classification of full-text documents, simulating the task of curators of the Mouse Genome Informatics (MGI) system and consisting of three subtasks. One subtask of the categorization task required the triage of articles likely to have experimental evidence warranting the assignment of GO terms, while the other two subtasks were concerned with the assignment of the three top-level GO categories to each paper containing evidence for these categories.

**Results:**

The track had 33 participating groups. The mean and maximum utility measure for the triage subtask was 0.3303, with a top score of 0.6512. No system was able to substantially improve results over simply using the MeSH term *Mice*. Analysis of significant feature overlap between the training and test sets was found to be less than expected. Sample coverage of GO terms assigned to papers in the collection was very sparse. Determining papers containing GO term evidence will likely need to be treated as separate tasks for each concept represented in GO, and therefore require much denser sampling than was available in the data sets.

The annotation subtask had a mean F-measure of 0.3824, with a top score of 0.5611. The mean F-measure for the annotation plus evidence codes subtask was 0.3676, with a top score of 0.4224. Gene name recognition was found to be of benefit for this task.

**Conclusion:**

Automated classification of documents for GO annotation is a challenging task, as was the automated extraction of GO code hierarchies and evidence codes. However, automating these tasks would provide substantial benefit to biomedical curation, and therefore work in this area must continue. Additional experience will allow comparison and further analysis about which algorithmic features are most useful in biomedical document classification, and better understanding of the task characteristics that make automated classification feasible and useful for biomedical document curation. The TREC Genomics Track will be continuing in 2005 focusing on a wider range of triage tasks and improving results from 2004.

## Background

Because of the growing size and complexity of the biomedical literature, there is increasing effort devoted to structuring knowledge in databases. One of the many key efforts is to annotate the function of genes. To facilitate this, the research community has come together to develop the Gene Ontology (GO, ) [1], a large, controlled vocabulary based on three axes or hierarchies:

• Molecular function (MF) – the activity of the gene product at the molecular (biochemical) level, e.g. protein binding

• Biological process (BP) – the biological activity carried out by the gene process, e.g., cell differentiation

• Cellular component (CC) – where in the cell the gene product functions, e.g., the nucleus

A major use of the GO has been to annotate the genomes of organisms used in biological research. The annotations are often linked to other information, such as literature, the gene sequence, the structure of the resulting protein, etc. An increasingly common approach is to develop "model organism databases" that bring together all the information for a specific organism into an easy to use format. Some of the better-known model organism databases include those devoted to the mouse (Mouse Genome Informatics, MGI, ) and the yeast (Saccharomyces Genome Database, SGD, ). These databases require extensive human effort for curation and annotation, which is usually done by PhD-level researchers. These curators could be aided substantially by high-quality information tools, including automated document categorization systems.

In the categorization task, using data extracted for us from the MGI databases by the MGI staff, we simulated two of the classification activities carried out by human annotators for the MGI system: a triage task and two simplified variations of MGI's annotation task. Systems were required to classify full-text documents from a two-year span (2002–2003) of three journals, with the first year's (2002) documents comprising the training data and the second year's (2003) documents making up the test data.

One of the goals of MGI is to provide structured, coded annotation of gene function from the biological literature. Human curators identify genes and assign GO codes about gene function with another code describing the type of experimental evidence supporting assignment of the GO code. The huge amount of literature requiring curation creates a challenge for MGI, as their resources are not unlimited. As such, they employ a three-step process to identify the papers most likely to describe gene function:

### 1. About mouse

The first step is to identify articles about mouse genomics biology. The full text of articles from several hundred journals is searched for the words *mouse*, *mice*, or *murine*. Articles passing this step are further analyzed for inclusion in MGI. At present, articles are searched in a Web browser one at a time because full-text searching is not available for all of the journals included in MGI.

### 2. Triage

The second step is to determine whether the identified articles should be sent for curation. MGI curates articles not only for GO terms, but also for other aspects of biology, such as gene mapping, gene expression data, phenotype description, and more. For GO curation, MGI strives to select only the articles that contain evidence supporting assignment of a GO code to a specific gene. The goal of this triage process is to limit the number of articles sent to human curators for more exhaustive and specific analysis. Articles that pass this step go into the MGI system with tags for GO, gene mapping, embryological expression, etc. The rest of the articles are not entered into MGI. Our triage task involved correctly classifying which documents had been selected for GO annotation in this process.

### 3. Annotation

The third step is the actual curation with GO terms. Curators identify genes for which there is experimental evidence to warrant assignment of GO codes. Those GO codes are assigned, along with an additional code for each GO code indicating the type of experimental evidence. There can more than one gene assigned specific GO codes in a given paper, and there can be more than one GO code assigned to a gene. In general, and in our collection, there is only one evidence code per GO code assignment per paper. Our annotation task involved a simplification of this annotation step. The goal of this task was not to select the actual GO term, but rather to automatically select the one or more GO hierarchies (molecular function, biological process, or cellular component) from which terms had been selected to annotate the gene for the article. Systems attempting to automate this step must both identify the individual genes, perhaps using named entity recognition techniques [2], as well as the corresponding GO code hierarchy. For the secondary subtask, systems must identify the evidence type code as well.

A shorter, preliminary version of this paper lacking much of the analysis and discussion presented here was posted originally online at "".

## Methods

The documents for the categorization task consisted of articles from three journals over two years, reflecting the full-text documents we were able to obtain from Highwire Press . Highwire is a "value added" electronic publisher of scientific journals. Most journals in their collection are published by professional associations, with the copyright remaining with the associations. Highwire originally began with biomedical journals, but in recent years has expanded into other disciplines. They have also supported IR (information retrieval) and related research by acting as an intermediary between consenting publishers and information systems research groups who want to use their journals, such as the TREC Genomics Track.

The journals available and used by our track this year were *Journal of Biological Chemistry *(JBC), *Journal of Cell Biology *(JCB), and *Proceedings of the National Academy of Science *(PNAS). These journals have a good proportion of mouse genome articles. Each of the papers from these journals was provided in SGML format based on Highwire's Document Type Definition (DTD). We used articles from the year 2002 for training data and from 2003 for test data. The documents for the categorization tasks came from a subset of articles having the words *mouse*, *mice *or *murine *as described above. We created a crosswalk file (look-up table) that matched an identifier for each Highwire article (its file name) and its corresponding PubMed ID (PMID). Table 1 shows the total number of articles in each journal and the number in each journal included in the subset used by the track. The SGML training document collection was 150 megabytes in size compressed and 449 megabytes uncompressed. The SGML test document collection was 140 megabytes compressed and 397 megabytes uncompressed.

**Table 1:** Number of papers total and available in the mouse, mus, or murine subset.

**Journal**	**2002 papers – total, subset**	**2003 papers – total, subset**	**Total papers – total, subset**
JBC	6566, 4199	6593, 4282	13159, 8481
JCB	530, 256	715, 359	1245, 615
PNAS	3041, 1382	2888, 1402	5929, 2784
Total papers	10137, 5837	10196, 6043	20333, 11880

**Figure 1:**
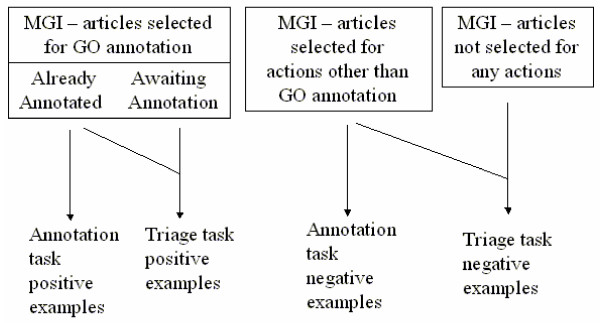
**Document grouping. **Grouping of documents for categorization subtasks.

Since MGI annotation lags behind article publication, a substantial number of papers had been selected for annotation but not yet annotated. From the standpoint of the triage subtask, we wanted to use all of these articles as positive examples, since they all were selected for GO annotation. However, we could not use the articles not yet annotated for the annotation hierarchy task, since we did not have the annotations. We also needed a set of negative examples for the annotation hierarchy task and chose to use articles selected for action by MGI for other (i.e., non-GO annotation) actions. These negative examples contain information about mouse research but do not include evidence for assignment of a GO code. Figure 1 shows the groups of documents and how they were assigned into being positive and negative examples for the subtasks.

### Triage subtask

The goal of the triage subtask was to correctly identify papers that were deemed to have experimental evidence warranting annotation with GO codes. Positive examples included papers designated for GO annotation by MGI. As noted above, some of these papers had not yet been annotated. Negative examples were all papers not designated for GO annotation in the operational MGI system. For the training data (2002), there were 375 positive examples, and 5462 negative examples. For the test data (2003), there were 420 positive examples, and 5623 negative examples. See Table 2. It should also be noted that the MGI system is, like most operational databases, continuously updated, so the data for the track represented a snapshot of the database obtained in May, 2004.

**Table 2:** Data set positive and negative sample counts.

**Data Set**	**Positive Samples**	**Negative Samples**	**Total Samples**
Training (year 2002)	375	5462	5837
Test (year 2003)	420	5623	6043

The evaluation measure for the triage task was the utility measure often applied in text categorization research and used by the former TREC Filtering Track. This measure contains coefficients for the utility of retrieving relevant and non-relevant documents. We used a version that was normalized by the best possible score:

U_norm _= U_raw _/ U_max_

where U_norm _was the normalized score, U_raw _the raw score, and U_max _the best possible score.

The coefficients for the utility measure were derived as follows. For a test collection of documents to categorize, U_raw _is calculated as:

U_raw _= (u_r _* relevant-docs-retrieved) + (u_nr _* non-relevant-docs-retrieved)

where:

• u_r _= relative utility of relevant document

• u_nr _= relative utility of non-relevant document

We used values for u_r _and u_nr _that were driven by boundary cases for different results. In particular, we thought it was important that the measure have the following characteristics:

• Completely perfect prediction: U_norm _= 1

• All documents designated positive (triage everything): 1 > U_norm _> 0

• All documents designated negative (triage nothing): U_norm _= 0

• Completely imperfect prediction (all predictions wrong): U_norm _< 0

We fixed u_nr _at -1 as is typically done. In order to achieve the above boundary cases, we had to set u_r _> 1. The ideal approach would have been to interview MGI curators and use decision-theoretic approaches to determine their utility. However, time and resource constraints did not allow this. We decided that the triage-everything approach must have a higher score than the triage-nothing approach, since the current practice at MGI is to examine (triage) everything for GO evidence and that practice certainly has value to MGI and the many users of its database. Triaging nothing would result in no GO evidence being curated. Since the current process has value, but also leaves much room for improvement in efficiency, we estimated that a U_norm _in the range of 0.25–0.3 for the triage-everything condition would be appropriate. Solving for the above boundary cases with U_norm_~0.25–0.3 for that case, we obtained a value for u_r_~20. To keep calculations simple, we choose a value of u_r _= 20. 3 shows the value of U_norm _for all four boundary cases.

**Table 3:** Boundary cases for utility measure of triage task for training and test data.

**Situation**	** U_norm_- Training**	**U_norm_- Test**
Completely perfect prediction	1.0	1.0
Triage everything	0.27	0.33
Triage nothing	0	0
Completely imperfect prediction	-0.73	-0.67

The measure U_max _was calculated by assuming all relevant documents were retrieved and no non-relevant documents were retrieved, i.e., completely perfect prediction and U_max _= u_r _* all-relevant-docs-retrieved.

Thus, for the training data,

U_raw _= (20 * relevant-docs-retrieved) - nonrelevant-docs-retrieved

U_max _= 20 * 375 = 7500

U_norm _= [(20 * relevant-docs-retrieved) - nonrelevant-docs-retrieved] / 7500

Likewise, for the test data,

U_raw _= (20 * relevant-docs-retrieved) - nonrelevant-docs-retrieved

U_max _= 20 * 420 = 8400

U_norm _= [(20 * relevant-docs-retrieved) - nonrelevant-docs-retrieved] / 8400

### Annotation subtasks

The primary goal of annotation subtask was, given an article and gene name, to correctly identify which of the GO hierarchies (also called domains) had terms within them that were annotated by the MGI curators. Note that the goal of this task was not to select the actual GO term, but rather to select the one or more GO hierarchies (molecular function, biological process, or cellular component) from which terms had been selected to annotate the gene for the article. Papers that were annotated had terms from one to three hierarchies.

For negative examples, we used 555 papers that had a gene name assigned but were used for other purposes by MGI. As such, these papers had no GO annotations. These papers did, however, have one or more genes assigned by MGI for the other annotation purposes.

A secondary subtask was to identify the correct GO evidence code that went with the hierarchy code. These evidence codes distinguish the type of evidence that the article provides for assigning the GO code, such as IDA (inferred from direct assay), or IMP (inferred from mutant phenotype). Only two groups took part in this subtask. Table 4 shows the contents and counts of the data files for this subtask. For the training data, there were a total of 504 documents that were either positive (one or more GO terms assigned) or negative (no GO terms assigned) examples. From these documents, a total of 1291 genes had been assigned by MGI. (The Genes file contained the MGI identifier, the gene symbol, and the gene name. It did not contain any other synonyms.) There were 1418 unique possible document-gene pairs in the training data. The data from the first three rows of Table 4 differ from the rest in that they contained data merged from positive and negative examples. These were what would be used as input for systems to nominate GO domains or the GO domains plus their evidence codes per the annotation task. When the test data were released, these three files were the only ones that were provided.

**Table 4:** Data file contents and counts for annotation hierarchy subtasks.

**File contents**	**Training data count**	**Test data count**
Documents – PMIDs	504	378
Genes – Gene symbol, MGI identifier, and gene name for all used	1294	777
Document gene pairs – PMID-gene pairs	1418	877
Positive examples – PMIDs	178	149
Positive examples – PMID-gene pairs	346	295
Positive examples – PMID-gene-domain tuples	589	495
Positive examples – PMID-gene-domain-evidence tuples	640	522
Positive examples – all PMID-gene-GO-evidence tuples	872	693
Negative examples – PMIDs	326	229
Negative examples – PMID-gene pairs	1072	582

For the positive examples in the training data, there were 178 documents and 346 document-gene pairs. There were 589 document-gene name-GO domain tuples (out of a possible 346 * 3 = 1038). There were 640 document-gene name-GO domain-evidence code tuples. A total of 872 GO plus evidence codes had been assigned to these documents. For the negative examples, there were 326 documents and 1072 document-gene pairs. This meant that systems could possibly assign 1072*3 = 3216 document-gene name-GO domain tuples. Note that MGI evidence codes refer to the type of evidence, not the specific thing that there is evidence for. Some documents contained evidence of more than one type for a gene and GO domain.

The evaluation measures for the annotation subtasks were based on the notion of identifying tuples of data. Given the article and gene, systems designated one or both of the following tuples:

• <article, gene, GO hierarchy code>

• <article, gene, GO hierarchy code, evidence code>

We employed a global recall, precision, and F-measure evaluation measure for each subtask:

• Recall = number of tuples correctly identified / number of correct tuples

• Precision = number of tuples correctly identified / number of tuples identified

• F = (2 * recall * precision) / (recall + precision)

For the training data, the total number of correct <article, gene, GO hierarchy code> tuples was 589, while the total number of correct <article, gene, GO hierarchy code, evidence code> tuples was 640.

Examples of the required submission format for each subtask are shown in 5.

**Table 5:** Example required submission format for each task.

Task	**Tab Delimited Submission Entry Format**					
Triage	Format: <TASK>	<PMID>				<TAG>
	Example: triage	Example: 12213961				Example: OHSU_TR

Annotation hierarchy	Format: <TASK>	<PMID>	<GENE>	<HIERARCHY>		<TAG>
	Example: annhi	12213961	Stat4	BP		OHSU_AH

Annotation hierachy plus evidence	Format: <TASK>	<PMID>	<GENE>	<HIERARCHY>	<EVIDENCE CODE>	<TAG>
	Example: annhiev	12213961	Stat4	BP	IDA	OHSU_AHPE

## Results

There were 98 runs submitted from 20 groups for the categorization task. These were distributed across the subtasks of the categorization task as follows: 59 for the triage subtask, 36 for the annotation hierarchy subtask, and three for the annotation hierarchy plus evidence code subtask.

### Triage subtask

The results of the triage subtask are shown in 6. A variety of groups used classifiers based on many different machine learning techniques. For example, the group from Rutgers University used a classifier based on a Bayesian Logistic Regression model [3], the group from Patolis Corporation used an SVM based classifier [4], and our group from OHSU used a modified voting perceptron classification algorithm [5]. The higher scoring runs tended to make use of MeSH terms in some fashion. The best performing run came from Rutgers, using the MEDLINE record, weighting, and filtering by the MeSH term *Mice *[3]. They achieved a Unorm of 0.6512.

**Table 6:** Triage subtask runs, sorted by utility.

**Run**	**Group (reference)**	**Precision**	**Recall**	**F-score**	**Utility**
dimacsTfl9d	rutgers.dayanik [3]	0.1579	0.8881	0.2681	0.6512
dimacsTl9mhg	rutgers.dayanik [3]	0.1514	0.8952	0.259	0.6443
dimacsTfl9w	rutgers.dayanik [3]	0.1553	0.8833	0.2642	0.6431
dimacsTl9md	rutgers.dayanik [3]	0.173	0.7952	0.2841	0.6051
pllsgen4t3	patolis.fujita [4]	0.149	0.769	0.2496	0.5494
pllsgen4t4	patolis.fujita [4]	0.1259	0.831	0.2186	0.5424
pllsgen4t2	patolis.fujita [4]	0.1618	0.7238	0.2645	0.5363
pllsgen4t5	patolis.fujita [4]	0.174	0.6976	0.2785	0.532
pllsgen4t1	patolis.fujita [4]	0.1694	0.7024	0.273	0.5302
GUCwdply2000	german.u.cairo [11]	0.151	0.719	0.2496	0.5169
KoikeyaTri1	u.tokyo (none)	0.0938	0.9643	0.171	0.4986
OHSUVP	ohsu.hersh [5]	0.1714	0.6571	0.2719	0.4983
KoikeyaTri3	u.tokyo (none)	0.0955	0.9452	0.1734	0.4974
KoikeyaTri2	u.tokyo (none)	0.0913	0.9738	0.167	0.4893
NLMT2SVM	nlm.umd.ul [12]	0.1286	0.7333	0.2188	0.4849
dimacsTl9w	rutgers.dayanik [3]	0.1456	0.6643	0.2389	0.4694
nusbird2004c	mlg.nus [13]	0.1731	0.5833	0.267	0.444
lgct1	indiana.u.seki [7]	0.1118	0.7214	0.1935	0.4348
OHSUNBAYES	ohsu.hersh [5]	0.129	0.6548	0.2155	0.4337
NLMT2BAYES	nlm.umd.ul [12]	0.0902	0.869	0.1635	0.4308
THIRcat04	tsinghua.ma [14]	0.0908	0.7881	0.1628	0.3935
GUClin1700	german.u.cairo [11]	0.1382	0.5595	0.2217	0.3851
NLMT22	nlm.umd.ul [12]	0.1986	0.481	0.2811	0.3839
NTU2v3N1	ntu.chen [15]	0.1003	0.6905	0.1752	0.381
NLMT21	nlm.umd.ul [12]	0.195	0.4643	0.2746	0.3685
GUCply1700	german.u.cairo [11]	0.1324	0.5357	0.2123	0.3601
NTU3v3N1	ntu.chen [15]	0.0953	0.6857	0.1673	0.3601
NLMT2ADA	nlm.umd.ul [12]	0.0713	0.9881	0.133	0.3448
lgct2	indiana.u.seki [7]	0.1086	0.581	0.183	0.3426
GUClin1260	german.u.cairo [11]	0.1563	0.469	0.2345	0.3425
THIRcat01	tsinghua.ma [14]	0.1021	0.6024	0.1746	0.3375
NTU4v3N1416	ntu.chen [15]	0.0948	0.6357	0.165	0.3323
THIRcat02	tsinghua.ma [14]	0.1033	0.5571	0.1743	0.3154
biotext1trge	u.cberkeley.hearst [16]	0.0831	0.7	0.1486	0.3139
GUCply1260	german.u.cairo [11]	0.1444	0.4333	0.2167	0.305
OHSUSVMJ20	ohsu.hersh [5]	0.2309	0.3524	0.279	0.2937
biotext2trge	u.cberkeley.hearst [16]	0.095	0.5548	0.1622	0.2905
THIRcat03	tsinghua.ma [14]	0.0914	0.55	0.1567	0.2765
THIRcat05	tsinghua.ma [14]	0.1082	0.4167	0.1718	0.245
biotext3trge	u.cberkeley.hearst [16]	0.1096	0.4024	0.1723	0.2389
nusbird2004a	mlg.nus [13]	0.1373	0.3357	0.1949	0.2302
nusbird2004d	mlg.nus [13]	0.1349	0.2881	0.1838	0.1957
nusbird2004b	mlg.nus [13]	0.1163	0.3	0.1677	0.1861
eres2	u.edinburgh.sinclair [17]	0.1647	0.231	0.1923	0.1724
biotext4trge	u.cberkeley.hearst [16]	0.1271	0.2571	0.1701	0.1688
emet2	u.edinburgh.sinclair [17]	0.1847	0.2071	0.1953	0.1614
epub2	u.edinburgh.sinclair [17]	0.1729	0.2095	0.1895	0.1594
nusbird2004e	mlg.nus [13]	0.136	0.231	0.1712	0.1576
geneteam3	u.hospital.geneva [18]	0.1829	0.1833	0.1831	0.1424
edis2	u.edinburgh.sinclair [17]	0.1602	0.1857	0.172	0.137
wdtriage1	indiana.u.yang [19]	0.202	0.1476	0.1706	0.1185
eint2	u.edinburgh.sinclair [17]	0.1538	0.1619	0.1578	0.1174
NTU3v3N1c2	ntu.chen [15]	0.1553	0.1357	0.1449	0.0988
geneteam1	u.hospital.geneva [18]	0.1333	0.1333	0.1333	0.09
geneteam2	u.hospital.geneva [18]	0.1333	0.1333	0.1333	0.09
biotext5trge	u.cberkeley.hearst [16]	0.1192	0.1214	0.1203	0.0765
TRICSUSM	u.sanmarcos [20]	0.0792	0.1762	0.1093	0.0738
IBMIRLver1	ibm.india (none)	0.2053	0.0738	0.1086	0.0595
EMCTNOT1	tno.kraaij [21]	0.2	0.0143	0.0267	0.0114
Mean		0.1381	0.5194	0.1946	0.3303
MeSH *Mice*	rutgers.dayanik [3]	0.1502	0.8929	0.2572	0.6404

**Figure 2:**
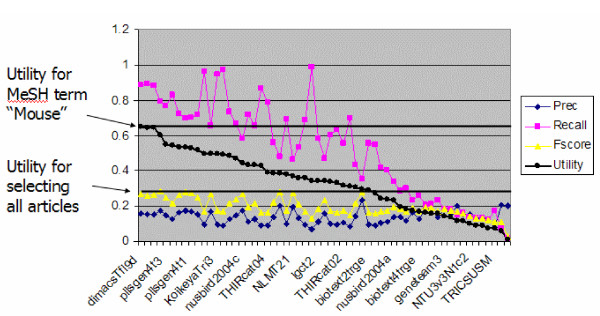
**Triage subtask. **Triage subtask runs sorted by Unorm score. The Unorm for the MeSH term *Mice *as well as for selecting all articles as positive is shown.

However, this group also noted that the MeSH term *Mice *alone scored better than all but the single top run, with a Unorm of 0.6404. This meant that no other approach was better able to classify documents for triage than simply using the MeSH term *Mice *from the MEDLINE record. Of course, this run only achieved a precision of about 15% (with a recall of 89%), so this feature is far from a perfect predictor. All of the triage subtask results are shown graphically in Figure 2, along with the utility for the MeSH term *Mice *and the decision to select all articles.

Because of these results we further analyzed the text collections, comparing the features identified as strong predictors in the training data (papers from the year 2002) with those in the test data (papers from the year 2003). One of the important issues in applying text classification systems to documents of interest to curators and annotators is how well the available training data represents the documents to be classified.

When classifying a biomedical text, the available training documents must have been written before the text to be classified. This is required for the TREC tasks to realistically simulate automation of the triage task of the GO curators. Papers written after a given article would not be available to the system for training prior to classifying that article. However, by its very nature the field of science changes over time, as does the language used to describe it. How rapidly the written literature of science changes has a direct influence on the development of biomedical text classification systems in terms of how features are generated and chosen, how often the systems need to be retrained, how large the training increment should be, and may effect the maximum performance that can be expected out of these systems.

We wanted to begin to understand this potentially important issue of *terminological drift *in the biomedical literature. In order to measure how well the features chosen from the training collection represented the information important in classifying the document in the test collection, we performed identical feature generation and selection processing on the training and test collections, including stemmed and stopped words, Chi-square feature selection at an alpha of 0.025, and inclusion of MeSH terms in the potential feature set. The process generated a set of 1885 features on the training collection and 1899 significant features on the test collection. We then measured how well the training collection feature set represented the test collection feature set by computing similarity metrics between the two sets [6]. The Dice similarity coefficient was 0.2489, the Jaccard similarity was 0.1422, cosine similarity was 0.2489, and the overlap measure was 0.2499. All similarity measures show a low level of similarity between the two sets.

We performed equivalent similarity measures on the individual word frequencies in the training and test collection, filtered out common English words as before, and sorted the words most frequent to least frequent for both sets. Computing similarity measures between the top 100, 1000, and 10,000 words in both sets showed consistently high similarity measures, with the maximum being the Dice similarity coefficient of 0.9618 at 100 words, and the minimum being a Jaccard similarity of 0.9232 at 10,000 words.

**Figure 3:**
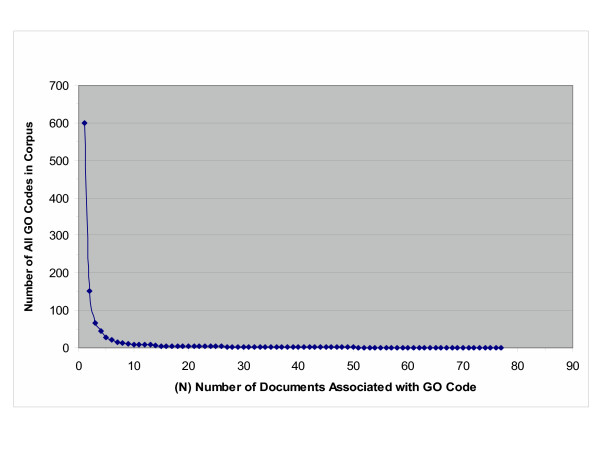
**Number of GO codes by document frequency. **This graph shows the number of GO codes at increasing levels of frequency that appear in the combined (test + training) corpus.

We were also interested in how well the GO codes assigned to the documents using training and test collections overlapped. Figure 3 shows a plot of the number of GO codes in the combined (training plus testing) corpus, as a function of the number of documents associated with each of those GO codes. As can be readily seen, the vast majority of GO codes associated with documents in the corpus are associated with only a single document in the corpus (448 out of 599), while 90% of GO codes appearing in the corpus are associated with two or less documents.

**Figure 4:**
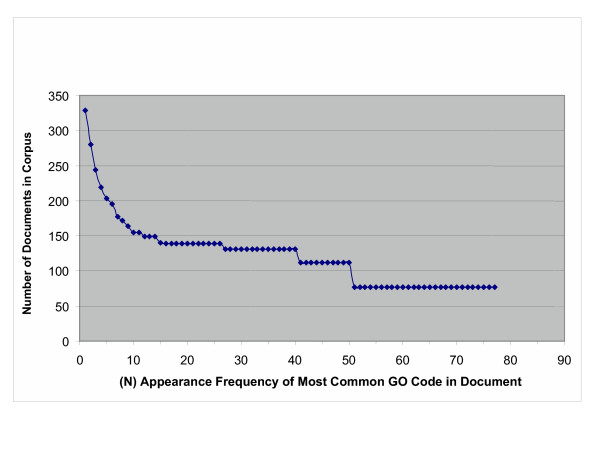
**Number of documents with frequency of most common GO code. **This graph shows the number of combined corpus documents having a most common GO code whose frequency is given on the x-axis.

Recall that a paper should be triaged positive for GO if there is evidence for any of the topics contained in the 20,000 GO codes. A paper may contain evidence for more than one GO code, but given the limited size of the training set, a paper is more likely to be classified as positive for GO if it contains evidence for a common GO code rather than a rare one. Figure 4 analyzes this situation in the combined corpus. The figure displays the number of documents in the corpus, whose most common associated GO code is given (by the GO codes frequency in the corpus) on the x-axis.

It is clear that a significant number of documents (48 out of 328, about 15%) have a "most common" GO code that appears only once in the entire corpus. More than half of the documents have a most common GO code that appears less than 10 times in the entire corpus.

### Annotation hierarchy subtask

**Figure 5:**
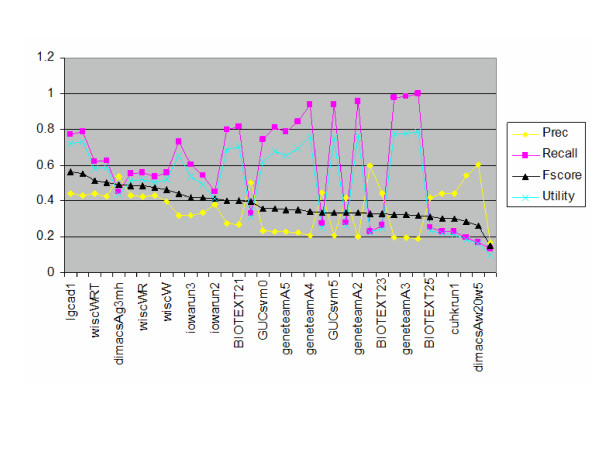
**Annotation hierarchy subtask. **Annotation hierarchy subtask results sorted by F-score.

The annotation hierarchy subtask results are shown in 7, while the annotation hierarchy subtask plus evidence code results are shown in Table 8. The primary evaluation measure for this task was the F-measure. Due to there only being a single measure per run, we were unable to perform comparative statistics. Figure 5 shows the annotation hierarchy subtask results graphically.

**Table 7:** Annotation hierarchy subtask, sorted by F-score.

**Run**	**Group (reference)**	**Precision**	**Recall**	**F-score**
lgcad1	indiana.u.seki [7]	0.4415	0.7697	0.5611
lgcad2	indiana.u.seki [7]	0.4275	0.7859	0.5537
wiscWRT	u.wisconsin [8]	0.4386	0.6202	0.5138
wiscWT	u.wisconsin [8]	0.4218	0.6263	0.5041
dimacsAg3mh	rutgers.dayanik [3]	0.5344	0.4545	0.4913
NLMA1	nlm.umd.ul [12]	0.4306	0.5515	0.4836
wiscWR	u.wisconsin [8]	0.4255	0.5596	0.4834
NLMA2	nlm.umd.ul [12]	0.427	0.5374	0.4758
wiscW	u.wisconsin [8]	0.3935	0.5596	0.4621
KoikeyaHi1	u.tokyo (none)	0.3178	0.7293	0.4427
iowarun3	u.iowa [22]	0.3207	0.6	0.418
iowarun1	u.iowa [22]	0.3371	0.5434	0.4161
iowarun2	u.iowa [22]	0.3812	0.4505	0.413
BIOTEXT22	u.cberkeley.hearst [16]	0.2708	0.796	0.4041
BIOTEXT21	u.cberkeley.hearst [16]	0.2658	0.8141	0.4008
dimacsAl3w	rutgers.dayanik [3]	0.5015	0.3273	0.3961
GUCsvm0	german.u.cairo [11]	0.2372	0.7414	0.3595
GUCir50	german.u.cairo [11]	0.2303	0.8081	0.3584
geneteamA5	u.hospital.geneva [18]	0.2274	0.7859	0.3527
GUCir30	german.u.cairo [11]	0.2212	0.8404	0.3502
geneteamA4	u.hospital.geneva [18]	0.209	0.9354	0.3417
BIOTEXT24	u.cberkeley.hearst [16]	0.4452	0.2707	0.3367
GUCsvm5	german.u.cairo [11]	0.2052	0.9354	0.3366
cuhkrun3	chinese.u.hongkong (none)	0.4174	0.2808	0.3357
geneteamA2	u.hospital.geneva [18]	0.2025	0.9535	0.334
dimacsAabsw1	rutgers.dayanik [3]	0.5979	0.2283	0.3304
BIOTEXT23	u.cberkeley.hearst [16]	0.4437	0.2626	0.3299
geneteamA1	u.hospital.geneva [18]	0.1948	0.9778	0.3248
geneteamA3	u.hospital.geneva [18]	0.1938	0.9798	0.3235
GUCbase	german.u.cairo [11]	0.1881	1	0.3167
BIOTEXT25	u.cberkeley.hearst [16]	0.4181	0.2525	0.3149
cuhkrun2	chinese.u.hongkong (none)	0.4385	0.2303	0.302
cuhkrun1	chinese.u.hongkong (none)	0.4431	0.2283	0.3013
dimacsAp5w5	rutgers.dayanik [3]	0.5424	0.1939	0.2857
dimacsAw20w5	rutgers.dayanik [3]	0.6014	0.1677	0.2622
iowarun4	u.iowa [22]	0.1692	0.1333	0.1492
Mean		0.3600	0.5814	0.3824

**Table 8:** Annotation hierarchy plus evidence code subtask, sorted by F-score.

**Tag**	**Group (reference)**	**Precision**	**Recall**	**F-score**
lgcab2	indiana.u.seki [7]	0.3238	0.6073	0.4224
lgcab1	indiana.u.seki [7]	0.3413	0.4923	0.4031
KoikeyaHiev1	u.tokyo (none)	0.2025	0.4406	0.2774
Mean		0.2892	0.5134	0.3676

In the annotation hierarchy subtask, the runs varied widely in recall and precision. The best runs, i.e., those with the highest F-measures, had medium levels of recall and precision. The top run came from Indiana University and used a variety of approaches, including a k-nearest-neighbor model, mapping terms to MeSH, using keyword and glossary fields of documents, and recognizing gene names [7]. Further post-submission runs raised their F-measure to 0.639. Across a number of groups, benefit was found from matching gene names appropriately. University of Wisconsin also found identifying gene names in sentences and modeling features in those sentences provided value [8].

## Discussion

The TREC 2004 Genomics Track categorization task featured a wide diversity of approaches, resulting in substantial variation across the results. Trying to discern the relative value of them is challenging, since few groups performed parameterized experiments or used common baselines.

The triage subtask was limited by the fact that using the MeSH term *Mice *assigned by the MEDLINE indexers was a better predictor of the MGI triage decision than anything else, including the complex feature extraction and machine learning algorithms of many participating groups. Some expressed concern that MGI might give preference to basing annotation decisions on maximizing coverage of genes instead of exhaustively cataloguing the literature, something that would be useful for users of its system but compromise the value of its data in tasks like automated article triage. We were assured by the MGI director (J. Blake, personal communication) that the initial triage decision for an article was made independent of the prior coverage of gene, even though priority decisions made later in the pipeline did take coverage into account. As such, the triage decisions upon which our data were based was sound from the standpoint of document classification.

The annotation decision was also not affected by this since the positive and negative samples were not exhaustive by design, that is, the data set for the annotation task did not include all article GO annotations made by MGI during this time period. The corpora do not need to be exhaustive for the results to be valid for this subtask; they must simply be correct for the training and test samples provided with GO hierarchies and evidence codes approximately evenly distributed.

Another concern about the MGI data was whether the snapshot obtained in mid-2004 was significantly updated by the time the track was completed. This was analyzed in early 2005, and it was indeed found that the number of PMIDs in the triage subtask had increased in size by about 10%, with a very small number of previously positive samples now negatively triaged (curators determined that these papers actually did not contain evidence for GO assignment). We re-ran our submitted methods on the updated data and obtained virtually identical results.

The major question for the triage subtask is why systems were unable to outperform the single MeSH term *Mice*. It should be noted that this term was far from perfect, achieving a recall of 89% but a precision of only 15%. So why did more elaborate systems not outperform this? There are a variety of possible explanations:

• MGI data is problematic – while MGI does some internal quality checking, they do not carry it out at the level that research groups would, e.g., with kappa scores.

• Our algorithms and systems are imperfect – we are unaware of or there do not exist better predictive feature sets and algorithms for this task.

• Our metrics may be problematic – is the factor = 20 in the utility formula appropriate? How do we determine a more appropriate means of computing utility that more accurately reflects the needs of the MGI curators?

• The terminological drift between the 2002 training corpus and the 2003 test corpus was large enough to reduce the effectiveness of all discriminating features except for the MeSH term *Mice*. Perhaps an online-style (incremental) training and evaluation method would be more appropriate than the batch method that we used here.

• The GO triage task is significantly more complex than previously studied document classification tasks. Much more data may be necessary to adequately train machine learning algorithms.

To some extent all of these explanations may play a factor, but the last is probably the dominant factor. The GO triage task appears significantly more difficult than previously studied biomedical document triage tasks. In the 2002 Knowledge Discovery and Data Mining (KDD) Challenge Cup, a task somewhat similar to the TREC triage task was organized around selection of papers about *Drosophila *(fruit fly) for curation in FlyBase, also using full text articles. Overall, analysis of the results showed that systems did quite well, with the best system achieving an F-measure of 78% on making yes/no decisions on papers, similar to the triage decision required in the TREC task [9].

The results of the TREC genomics track GO triage task appear significantly worse, with the best submission scoring a utility of 0.6512 and a corresponding F-score of about 27%. However, there are several important differences between the TREC and the KDD triage tasks, besides the obvious, but possibly important difference, that the KDD task focused on fly genomics and the TREC task on mouse. First of all, both the training and test collections for the KDD task had a relatively high proportion of positives (33% and 43%, respectively) as compared to the TREC task (6.5% and 7%). Furthermore, the TREC task used a utility measure heavily weighted towards high recall, while the KDD Cup used F-score, the balanced harmonic mean of recall and precision. Therefore the KDD measure did not take into account a curator preference for not missing many positive articles as we have done here, equally weighted correct prediction of positives and negatives, and had a proportion of positives approaching 50% in the test collection. These factors may have made scoring well on the KDD task easier compared to the TREC task.

Another difference between the TREC and KDD shared tasks may be even more important. The KDD FlyBase triage task was to "determine whether the paper meets the FlyBase gene expression curation criteria, and for each gene, indicate whether the full paper has experimental evidence for gene products (mRNA and/or protein)" [9]. Positive classification was determined solely on whether the full paper included experimental evidence linking genes to their products. The TREC task was to determine whether the paper contained evidence for assignment of GO codes, *any *GO code. Currently, there are about 20,000 different terms in the GO, in the areas of cellular component, molecular function, and biological process. This is clearly a much wider range of topics than simply gene transcription products, and makes the TREC GO task much more heterogeneous than the KDD task.

Figures 4 and 5 show that the sampling and coverage of GO terms in the training and testing sets, as well as the combined collection, is very sparse, both in terms of individual GO terms, and for papers containing evidence for common GO terms. With 20,000 different terms in the GO under three main headings, a great variety of different topic areas related to the individual GO terms may be present in our collection.

Each of these individual topics can be viewed as a separate yes/no classification task in itself. The GO triage categorization task may better be thought of as many subtasks, where classification of the presence/absence of each GO code is done individually, and the document is triaged for GO if classified as positive for any of the GO codes. But the individual GO codes are sampled very thinly. When the corpus is split into training and test collections, it is very likely that for most GO codes either the training or testing set will be either missing many codes, include only one document that is associated with a given code, or at best, very thinly sample the GO codes relevant for classifying a paper positive for the triage task. Therefore the corpus may contain many GO topics for which there are an inadequate number of cases to provide meaningful samples in both the test and training sets.

For about 85% of documents, the most common GO code associated with a document is found associated with two or more documents. Interestingly, this figure is very close to the recall of the best performing system for the GO triage task 88.8%, and may represent an upper limit on recall performance for this data set.

Combining the samples for each of the many GO topics together may result in the strong features for a given topic being obscured by the strong features in other topics, overwhelming any classification system with the resulting noise, with only features common to the majority of individual topics still predictive. It appears that the MeSH term *Mice *meets this description. The terminological drift showing a difference in significant features between the training and test collection may simply be due to the very sparse sampling of the range of GO topics over both years. This is substantiated by the data that the most common words (after stop word removal) were largely unchanged, but the statistically significant feature set changed quite a bit from the year 2002 to 2003.

All of the above lends support to the theory that the GO triage task is difficult because it contains many sub-problems which are very sparsely sampled. There aremany GO codes having only one associated document contained in the corpus, and there are many, many GO codes that are completely missing from the corpus. We believe that the triage subtask data represents an important task (i.e., document triage is valuable in a variety of biomedical settings, such as discerning the best evidence in clinical studies) and that these data provide the initial substrate for work to continue in this area. However, it appears that the corpushas to be much, much larger in order to support machine learning on the full range of GO codes for automated text classification on this specific task. Over time, MGI will collect vast amounts of data during the natural course of curating documents each year, but it may be a very long time before adequate numbers of samples are available for all GO codes. Selecting data specifically to train and test classification systems for identifying papers containing evidence for the most common GO codes and other, more specifically defined triage scenarios (such as embryological expression) may be more tractable tasks to address in the near term.

The annotation hierarchy task had lower participation, and the value of picking the correct hierarchy is unclear. However, there would be great value to systems that could perform automated GO annotation, even though the task is very challenging [10]. These results demonstrated value in identifying gene names and other controlled vocabulary terms in documents for this task.

## Conclusion

The automated classification of documents for GO annotation proved to be a challenging task. Automated extraction of GO hierarchy codes was even more challenging. This was the first year that the TREC Genomcs Track included a classification task, and so our understanding of the best way to approach these tasks for biomedical curation is just beginning. Current text classification systems are most often optimized for a balanced F-measure, where precision and recall are weighted evenly. However, the asymmetric utility measure used in the triage task was heavily weighted towards recall. This reflected the priorities of the document curators. It is likely that further experience optimizing for this type of utility measure will provide improved results.

Analysis of feature sets showed less correlation between statistically significant features in the training and test sets than expected. While this is most likely due to the sparse sampling of individual GO topics, there is currently insufficient evidence to determine the practical significance and generality of this, and whether this is a general problem for biomedical document classification.

While no approach was able to improve upon the triage performance of simply using the MeSH term *Mice*, this is likely due to the heterogeneity of the GO triage task and is unlikely to be the case for other, more specific biomedical document triage tasks. Additional research into other tasks will provide more information about the performance expectations for biomedical document classification. This task is likely not representative of document classification for biomedical curation tasks. The Mouse Genome Institute also curates articles for purposes other than GO annotation. Comparison with these tasks will provide further insight into the true potential of document classification for biomedical curation.

The TREC Genomics Track will be continuing in 2005. The categorization task will consist of selecting papers for a set of four triage categories relevant to MGI curation, including allele phenotypes, embryologic expression, and tumor biology as well as repeating the GO triage categorization task with updated data. It is hoped that the research community will be able to build on their experience from this year and present improved results in 2005. There is a large potential benefit to biomedical curation, and work in this area must continue to realize fully the advantages the automated biomedical document classification and text mining could bring to biomedical research.

## Competing interests

The author(s) declare that they have no competing interests.

## Authors' contributions

AC drafted the article, lead the OHSU submission for the TREC Genomics categorization task, and first suggested the asymmetric utility measure and boundary cases. WR chairs the TREC Genomics Track and initially suggested the track tasks described here. Both authors serve on the TREC Genomics Track steering committee and reviewed and approved the final manuscript.

## References

[B1] Anonymous (2004). The Gene Ontology (GO) database and informatics resource. Nucleic Acids Research.

[B2] Cohen AM, Hersh W (2005). A Survey of Current Work in Biomedical Text Mining. Briefings in Bioinformatics.

[B3] Dayanik A, Fradkin D, Genkin A, Kantor P, Madigan D, Lewis DD, Menkov V, Voorhees EM and Buckland LP (2004). DIMACS at the TREC 2004 Genomics Track: ; Gaithersburg, MD..

[B4] Fujita S, Voorhees EM and Buckland LP (2004). Revisiting again document length hypotheses - TREC 2004 Genomics Track experiments at Patolis: ; Gaithersburg, MD..

[B5] Cohen AM, Bhuptiraju RT, Hersh W, Voorhees EM and Buckland LP (2004). Feature generation, feature selection, classifiers, and conceptual drift for biomedical document triage: ; Gaithersburg, MD..

[B6] Dunham MH (2003). Data mining introductory and advanced topics.

[B7] Seki K, Costello JC, Singan VR, Mostafa J, Voorhees EM and Buckland LP (2004). TREC 2004 Genomics Track experiments at IUB: ; Gaithersburg, MD..

[B8] Settles B, Craven M, Voorhees EM and Buckland LP (2004). Exploiting zone information, syntactic rules, and informative terms in Gene Ontology annotation of biomedical documents: ; Gaithersburg, MD..

[B9] Yeh AS, Hirschman L, Morgan AA (2003). Evaluation of text data mining for database curation: lessons learned from the KDD Challenge Cup. Bioinformatics.

[B10] Hirschman L, Yeh A, Blaschke C, Valencia A (2005). Overview of BioCreAtIvE: critical assessment of information extraction for biology. BMC Bioinformatics.

[B11] Darwish K, Madkour A, Voorhees EM and Buckland LP (2004). The GUC goes to TREC 2004:  using whole or partial documents for retrieval and classification in the Genomics Track: ; Gaithersburg, MD..

[B12] Aronson AR, Demmer D, Humphrey SH, Ide NC, Kim W, Loane RR, Mork JG, Smith LH, Tanabe LK, Wilbur WJ, Xie N, Demner D, Liu H, Voorhees EM and Buckland LP (2004). Knowledge-intensive and statistical approaches to the retrieval and annotation of genomics MEDLINE citations: ; Gaithersburg, MD..

[B13] Zhang D, Lee WS, Voorhees EM and Buckland LP (2004). Experience of using SVM for the triage task in TREC 2004 Genomics Track: ; Gaithersburg, MD..

[B14] Li J, Zhang X, Zhang M, Zhu X, Voorhees EM and Buckland LP (2004). THUIR at TREC 2004: Genomics Track: ; Gaithersburg, MD..

[B15] Lee C, Hou WJ, Chen HH, Voorhees EM and Buckland LP (2004). Identifying relevant full-text articles for GO annotation without MeSH terms: ; Gaithersburg, MD..

[B16] Nakov PI, Schwartz AS, Stoica E, Hearst MA, Voorhees EM and Buckland LP (2004). BioText team experiments for the TREC 2004 Genomics Track: ; Gaithersburg, MD..

[B17] Sinclair G, Webber B, Voorhees EM and Buckland LP (2004). TREC Genomics 2004: ; Gaithersburg, MD..

[B18] Ruch P, Chichester C, Cohen G, Ehrler F, Fabry P, Marty J, Muller H, Geissbuhler A, Voorhees EM and Buckland LP (2004). Report on the TREC 2004 experiment:  Genomics Track: ; Gaithersburg, MD..

[B19] Yang K, Yu N, Wead A, LaRowe G, Li YH, Friend C, Lee Y, Voorhees EM and Buckland LP (2004). WIDIT in TREC 2004 Genomics, Hard, Robust and Web Tracks: ; Gaithersburg, MD..

[B20] Guillen R, Voorhees EM and Buckland LP (2004). Categorization of genomics text based on decision rules: ; Gaithersburg, MD..

[B21] Kraaij W, Raaijmakers S, Weeber M, Jelier R, Voorhees EM and Buckland LP (2004). MeSH based feedback, concept recognition and stacked classification for curation tasks: ; Gaithersburg, MD..

[B22] Eichmann D, Zhang Y, Bradshaw S, Qiu XY, Zhou L, Srinivasan P, Sehgal AK, Wong H, Voorhees EM and Buckland LP (2004). Novelty, question answering and genomics:  the University of Iowa response: ; Gaithersburg, MD..

